# Similarities of developmental gene expression changes in the brain between human and experimental animals: rhesus monkey, mouse, Zebrafish, and Drosophila

**DOI:** 10.1186/s13041-021-00840-4

**Published:** 2021-09-07

**Authors:** Ryuichi Nakajima, Hideo Hagihara, Tsuyoshi Miyakawa

**Affiliations:** Division of Systems Medical Science, Institute for Comprehensive Medical Science, Fujita Health University, 1-98 Dengakugakubo, Kutsukake-cho, Toyoake, Aichi 470-1192 Japan

**Keywords:** Gene expression, Development, Human, Rhesus monkey, Mouse, Zebrafish, Drosophila, RNA-seq

## Abstract

**Aim:**

Experimental animals, such as non-human primates (NHPs), mice, Zebrafish, and Drosophila, are frequently employed as models to gain insights into human physiology and pathology. In developmental neuroscience and related research fields, information about the similarities of developmental gene expression patterns between animal models and humans is vital to choose what animal models to employ. Here, we aimed to statistically compare the similarities of developmental changes of gene expression patterns in the brains of humans with those of animal models frequently used in the neuroscience field.

**Methods:**

The developmental gene expression datasets that we analyzed consist of the fold-changes and *P* values of gene expression in the brains of animals of various ages compared with those of the youngest postnatal animals available in the dataset. By employing the running Fisher algorithm in a bioinformatics platform, BaseSpace, we assessed similarities between the developmental changes of gene expression patterns in the human (*Homo sapiens*) hippocampus with those in the dentate gyrus (DG) of the rhesus monkey (*Macaca mulatta*), the DG of the mouse (*Mus musculus*), the whole brain of Zebrafish (*Danio rerio*), and the whole brain of Drosophila (*D. melanogaster*).

**Results:**

Among all possible comparisons of different ages and animals in developmental changes in gene expression patterns within the datasets, those between rhesus monkeys and mice were highly similar to those of humans with significant overlap *P*-value as assessed by the running Fisher algorithm. There was the highest degree of gene expression similarity between 40–59-year-old humans and 6–12-year-old rhesus monkeys (overlap *P*-value = 2.1 × 10^− 72^). The gene expression similarity between 20–39-year-old humans and 29-day-old mice was also significant (overlap *P* = 1.1 × 10^− 44^). Moreover, there was a similarity in developmental changes of gene expression patterns between 1–2-year-old Zebrafish and 40–59-year-old humans (Overlap *P-value* = 1.4 × 10^− 6^). The overlap *P*-value of developmental gene expression patterns between Drosophila and humans failed to reach significance (30 days Drosophila and 6–11-year-old humans; overlap *P*-value = 0.0614).

**Conclusions:**

These results indicate that the developmental gene expression changes in the brains of the rhesus monkey, mouse, and Zebrafish recapitulate, to a certain degree, those in humans. Our findings support the idea that these animal models are a valid tool for investigating the development of the brain in neurophysiological and neuropsychiatric studies.

**Supplementary Information:**

The online version contains supplementary material available at 10.1186/s13041-021-00840-4.

The use of animal models is invaluable for elucidating the underlying mechanisms of human physiology and pathology. Depending on many circumstances, such as the ethical requirements, the purpose of experiments, and efficiency of breeding, different species of experimental animals are employed for experiments. Among various types of animal models, non-human primates (NHPs) have the highest degree of genetic identity to humans, given their relatively recent evolutionary divergence from that of human beings [1, 2], and NHPs are employed in cases where primate-specific functions are the subject to study [3, 4], although the strictest ethical consideration is necessary. Mice also have similarities in gene expression patterns with humans [5]. They have advantages in rich genetic resources, their small size, ease of maintenance, and short life cycle, enabling the effective implementation of the diseases of humans [6–9]. Non-mammal animals, such as Zebrafish [10–17], and Drosophila [18–21], are also employed as experimental animals because of their technical advantages in maintenance, spatial requirements, fertility, genetic manipulation, and observation. In developmental neuroscience and the related fields using animal models, information about the developmental changes of the gene expression patterns in the brain of experimental animals and their correlation with human transcriptomics are important. Bakken et al. (2016) carried out a comprehensive transcriptional mapping of brain development in rhesus monkeys and compared the gene expression patterns in the frontal cortex with human’s and rat’s. They estimated the number of overlapping gene expressions in development and suggested that the number of overlapping genes between rhesus monkeys and humans was significantly higher than that between rats and humans using non-parametric statistical tests [22]. Gerstein et al. (2014) compared transcriptome across distant species and discovered that co-expression modules shared across humans, C-elegans, and Drosophila, many of which are enriched in developmental genes [23]. Howe et al. (2013) investigated genomic sequences between humans and Zebrafish and found that approximately 70 % of human genes have at least one obvious zebrafish orthologue [24]. However, quantitative information on the transcriptomic similarity across multiple species of animal models is still limited.

Here, using running Fisher analysis available in BaseSpace correlation engine (Illumina, San Diego, CA), we evaluated the similarity of developmental transcriptomes across different species (Additional file 3). We employed “overlap *P*-values” calculated from fold changes of gene expression, the *P*-values of the fold changes of the individual gene expressions, and their ranks [25]. This method allowed us to quantify the similarities in developmental changes of the gene expression pattern of brains between humans [26] and commonly-used animal models, consisting of rhesus monkeys [27], mice [28], Zebrafish [29], and Drosophila [21]. Dataset of the fold-changes and the *P*-values of gene expression of human that we analyzed consist of those from infants to elderly (6–12 months old, 1–5, 6–11, 12–19, 20–39, 40–59, and over 60 years old) in comparison with 0–5 months old infants. Likewise, those of mice from young to adult stages up to 6 months old (11, 14, 17, 21, 25, 29 days, and 6 months old) in comparison with young mice (8 days old), those of Zebrafish from the young to aged (Embryonic stage E5, E10, 3 months old, 1–2 years old) in comparison with E3, those of Drosophila from the 30 days old and the 60 days old in comparison with the 3 days old, were subjected to the present study.

We first compared the developmental gene expression changes between the human hippocampus [26] and the hippocampal DG of rhesus monkeys [27] available in BaseSpace. Among 21 combinations of the available datasets from different ages of humans and rhesus monkeys (Additional file 2: Table S1), there was the highest degree of gene expression similarity between those of 40–59-year-old humans and 6–12-year-old rhesus monkeys (Fig. 1A, overlap *P*-value = 2.1 × 10^− 72^), with 546 genes altered in both humans and rhesus monkeys. 503 genes out of those genes showed the same directional change in expression and, of these genes, 148 genes were upregulated (Fig. 1A, magenta bar; *P* = 4.1 × 10^− 41^), and 355 were downregulated (Fig. 1A, blue bar: *P* = 1.2 × 10^− 104^). Likewise, we compared similarities of the developmental gene expression changes of the human hippocampus [26] and hippocampal DG of mice that are available from Murano et al. (2019) [28]. Among the 49 combinations of datasets from different ages of humans and mice (Additional file 2: Table S1), the one between those of 20–39-year-old humans and 29-day old mice recorded the highest degree of gene expression overlap (Fig. 1B, overlap *P* value = 1.1 × 10^− 44^; 1474 genes altered in both humans and mice). The same directional change in gene expression was observed in 1072 genes, of which 419 genes were upregulated (Fig. 1B, magenta bar; *P* = 5.2 × 10^− 24^) and 653 downregulated (Fig. 1B, blue bar; *P* = 1.1 × 10^− 65^). Among 56 combinations of the datasets of the human hippocampus [26] and Zebrafish brain [29], 40-59-year-old humans and 1-2-year-old Zebrafish exhibited the highest degree of gene expression overlap (Fig. 1C, overlap *P-value* = 1.4 × 10^− 6^; 245 genes altered in both humans and Zebrafish). The same directional change in expression was observed in 161 genes, of which 40 were upregulated (Fig. 1C, magenta bar; *P* = 0.003) and 121 downregulated (Fig. 1C, blue bar; *P* = 7.7 × 10^− 11^). Finally, regarding the 14 combinations between the human hippocampus [26] and Drosophila brain [21] that we assessed, we identified the highest degree of gene expression overlap between those of 6–11-year-old humans and 30 days Drosophila (Fig. 1D, overlap *P-value* = 0.0614), with 303 genes altered in both humans and Drosophila. The same directional change in expression occurred in 66 genes, of which 15 genes were upregulated (Fig. 1D, magenta bar; *P* = 0.1915) and 51 downregulated (Fig. 1D, blue bar; *P* = 0.9218).

**Fig. 1:**
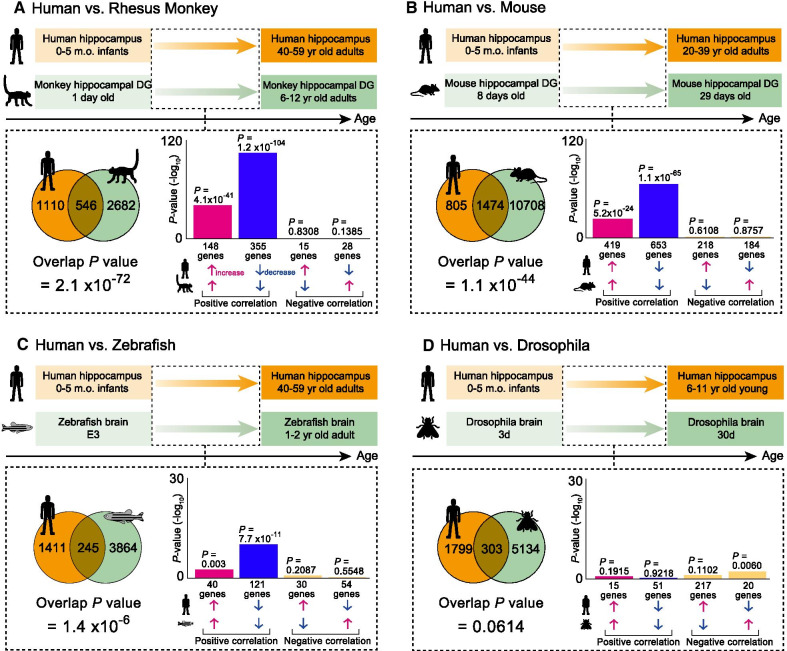
Similarities in temporal transcriptomics between brains of human and experimental animals: rhesus monkey, mouse, Zebrafish, and Drosophila. **A**–**D** The representative combination, which resulted in the lowest overlap *P-value* among all the data from developmental stages in each animal dataset (also see Additional file 2: Table S1), is indicated.  Comparison of gene expression patterns in the human hippocampus of 40–59-year-old adults compared with those of the hippocampal dentate gyrus of 6–12-year-old adult monkeys **(A)**. The Venn diagram indicates that there were 546 common genes whose expression levels significantly changed with aging in both hippocampi of 40–59-year-old adults and hippocampal DG of 6–12-year-old adult monkeys, and the overlap *P-value*, as assessed by running Fisher analysis, was 2.1 × 10^− 72^. The right bar graphs indicate that, within the 546 common genes, the expression of 148 genes increased and 355 genes decreased in both humans and monkeys (i.e., positive correlation); expression of 15 genes increased and decreased in humans and monkeys, respectively; and the expression of 28 genes decreased and increased in humans and monkeys, respectively (i.e., negative correlation). The overlap *P-value*s of these different types of correlations are also indicated above the corresponding bar graph. Likewise, gene expression patterns in the human hippocampus of 20–39-year-old adults compared with those of the hippocampal dentate gyrus of 29-day-old mice (**B**), gene expression patterns in the human hippocampus of 40–59-year-old adults compared with those of the brain of 1-2-year-old adult zebrafish (**C**), and gene expression patterns in the hippocampus of 6–11-year-old young humans compared with those of the 30-day old Drosophila brain (**D**), are indicated in the same manner with (**A**). *DG* dentate gyrus, *E* embryonic day, *m.o.* months old, *yr* year, *d* day

We have confirmed that rhesus monkeys, mice, and Zebrafish, which belong to deuterostomes, have developmental changes of gene expression patterns that are significantly similar to those of humans. In contrast, the developmental changes of the gene expression pattern of the brain of Drosophila, which belongs to protostomes, were not significantly correlated with those of humans. In *Caenorhabditis elegans* (*C. elegans*), which also belongs to protostomes, the developmental changes of the gene expression pattern of whole-body samples were weakly and negatively correlated with those of human brains (Additional file 1: Fig. S1 and Additional file 2: Table S6) [30]. Overall, the degrees of similarity between animal models and humans shown in this report tended to reflect their evolutionary distance from humans. It should be noted that we have conducted the analyses using publicly available data, of which subjected brain regions and developmental stages are not perfectly matched across the included species. For example, the sampling resolution and period of developmental stages differ across the animals, and the datasets of rhesus monkeys and mice do not contain the data from embryonic stages, while the datasets of humans and Zebrafish do. Also, the developmental transcriptomics data of *C. elegans* was obtained from whole-body, and so it is hard to directly compare its data with those from the other species evaluated in this study. Despite these limitations, this study indicates that gene expression patterns in rhesus monkeys, mice, and zebrafish match those in humans. These findings thus support the validity of these animal models for studying human brain development and development-related functions and dysfunctions.

## Supplementary Information

**Additional file 1: Figure S1.** Correlation of temporal transcriptomics between brains of humans and whole-bodies of *C. elegans*.

**Additional file 2: Table S1.** Matrix table of the overlap *P-values* of temporal transcriptomics between all the available ages of the brains of human and experimental animals: rhesus monkey, mouse, Zebrafish, and Drosophila. **Table S2.** Gene expression in the hippocampus of 40–59-year-old humans and in the DG of 6–12-year-old rhesus monkeys (fold change and rank; Fig. 1A). **Table S3.** Gene expression in the hippocampus of 20–39-year-old humans and in the DG of 629-day-old mice (fold change and rank; Fig. 1B). **Table S4.** Gene expression in the hippocampus of 40–59-year-old humans and in 1–2-year-old adult zebrafish brain (fold change and rank; Fig. 1C). **Table S5.** Gene expression in the hippocampus of 6–11-year-old young humans and in 30-day old Drosophila brain (fold change and rank; Fig. 1D). **Table S6.** Gene expression in the hippocampus of 6–11-year-old young humans and in 4 days old *C. elegans* (fold change and rank). **Table S7.** Top-40 of the correlating gene expression.

**Additional file 3.** Method for the calculation of overlap *P-value* by running Fisher analysis.

## Data Availability

The data that support the findings of this study are available as Additional files.

## Abbreviations

NHPs: Non-human primates
DG: Dentate gyrus
