# Supplementary material for: Similarities of developmental gene expression changes in the brain between human and experimental animals: rhesus monkey, mouse, Zebrafish, and Drosophila
Source: Mol Brain. 2021 Sep 7;14:135. doi: 10.1186/s13041-021-00840-4 (PMC8425040; doi:10.1186/s13041-021-00840-4)
Supplement: Supplementary file 1 — Additional file 1: Figure S1. Correlation of temporal transcriptomics between brains of humans and whole-bodies of C. elegans. [file 13041_2021_840_MOESM1_ESM.pdf]

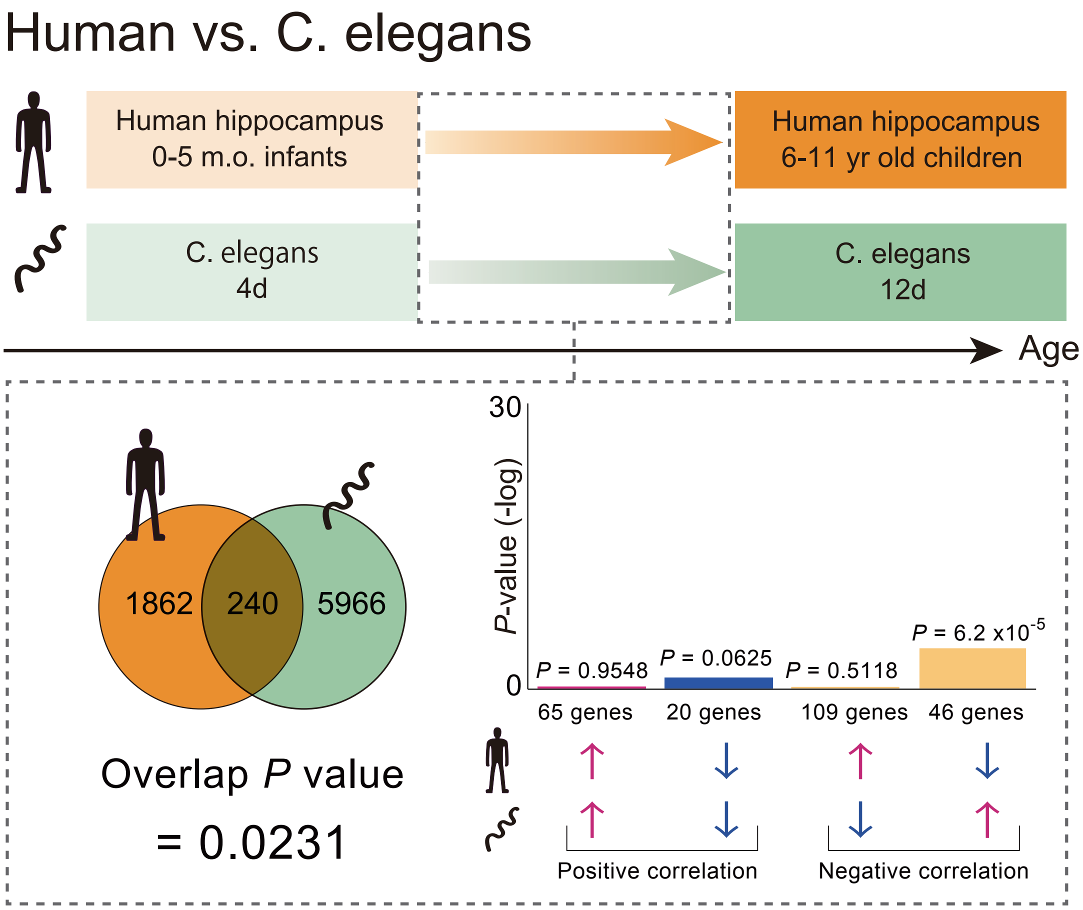

Supplementary Figure 1. Correlation of temporal transcriptomics between brains of humans and whole-bodies of C. elegans. The representative combination, which resulted in the lowest overlap *P-value* among all the data from developmental stages in C. elegans and humans (see Table S1), is indicated. Comparison of gene expression patterns in the human hippocampus of 6–11-year-old children compared with those of the whole-body of 12-day-old C. elegans. The Venn diagram indicates that there were 240 common genes whose expression levels significantly changed with aging in both hippocampi of 6–11-year-old children and whole-body of 12-day-old C. elegans, and the overlap *P-value* of 0.0231, as assessed by running Fisher analysis. The right bar graphs indicate that, within the 240 common genes, the expression of 65 genes increased and 20 genes decreased in both humans and C. elegans (i.e., positive correlation); expression of 109 genes increased and decreased in humans and C. elegans, respectively; and the expression of 46 genes decreased and increased in humans and C. elegans, respectively (i.e., negative correlation). The overlap *P-value*s of these different types of correlations are also indicated above the corresponding bar graph.
