# Supplementary material for: Similarities of developmental gene expression changes in the brain between human and experimental animals: rhesus monkey, mouse, Zebrafish, and Drosophila
Source: Mol Brain. 2021 Sep 7;14:135. doi: 10.1186/s13041-021-00840-4 (PMC8425040; doi:10.1186/s13041-021-00840-4)
Supplement: Supplementary file 3 — Additional file 3. Method for the calculation of overlap P-value by running Fisher analysis. [file 13041_2021_840_MOESM3_ESM.pdf]

SUPPLEMENTARY METHODS

The Supplementary Methods below were adopted from Murano et al. (2019) under the terms of Creative Commons Attribution 4.0 International License (CC BY)(1).

Computing overlap *P-values* of gene expression patterns in different datasets

BaseSpace (Illumina, San Diego, CA) was used to compare the signatures in publicly available microarray datasets with a signature provided by the user using a "Running Fisher" algorithm, as previously described (2–6). In brief, the overlap *P-value*, i.e., the direction of the correlation between two given gene expression datasets (*b1, b2*), and the *P-values* of the similarities between two subsets of gene expression datasets were calculated as follows:

Each gene signature set was rank-ordered according to the absolute fold-change value. Upregulated and downregulated genes were denoted by positive and negative signs, respectively, to indicate directionality. A directional subset was generated for each direction, such as *b1+, b1-, b2+,* and *b2-*.

Next, all subset pairs were identified as *b1Di*, *b2Dj*, where *Di* and *Dj* were the available directions (+ or −) in *b1* and *b2*, respectively. We applied the Running Fisher algorithm to each subset pair. The top-ranking genes in the first subset *b1Di* were collected as a group, *G*. The second subset *b2Dj* was scanned from top to bottom in rank order to identify each rank with a gene matching a member in group *G*. At each matching rank, *K*, the scanned portion of the second subset *b2Dj*, consisted of *N* genes. The overlap between group *G* and the *N* genes was defined as *M*. Fisher's exact test that was performed at rank *K* to evaluate the statistical significance of observing *M* overlaps between a set of size *G* and a set of size *N*, where the set of size *G* comes from platform *P1*. The set of size *N* comes from platform *P2*, given the sizes of *P1* and *P2* and the overlap between *P1* and *P2*. At the end of the scan, we retained the best *P-value* and applied multiple-hypothesis-testing correction factor. The negative log of the multiple-testing-corrected best *P-value* ($P_{b1Di\rightarrow b2Dj}$) was a score ($S_{b1Di\rightarrow b2Dj}$) for the subset pair. Here, the subscript *b1Di → b2Dj* indicates that *b1Di* was the first subset used to define the top genes *G,* and *b2Dj* was the second subset used for the scan.

$$S_{b1Di\rightarrow b2Dj} = -\ln P_{b1Di\rightarrow b2Dj} \qquad (1)$$

Next, we performed the Running Fisher algorithm in the reverse direction. The same procedure in this reverse direction produced another score $S_{b2Di\rightarrow b1Dj}$) for the same subset pair. The two scores were averaged to represent the magnitude of the similarity between the two subsets.

$$S_{b1Dib2Dj} = \frac{S_{b1Di\rightarrow b2Dj} + S_{b2Dj\rightarrow b1Di}}{2} \qquad (2)$$

The *P-value* ($P_{b1Dib2Dj}$) between *b1Di* and *b2Dj* was calculated using the following equation:

$$P_{b1Dib2Dj} = exp\ (-S_{b1Dib2Dj}) \qquad (3)$$

A positive sign was assigned to pairwise correlation scores (S_{b1+b2+} and S_{b1-b2-}) for a subset pair of the same direction (*b1+b2+*, *b1-b2-*), and a negative sign was assigned to pairwise correlation scores (S_{b1+b2-} and S_{b1-b2+}) for a subset pair of opposite directions (*b1+b2-*, *b1-b2+*). Then, we calculated the overall score (*Sb1b2*) between b1 and b2 from the correlation scores (S_{b1+b2+}, S_{b1-b2-}, S_{b1+b2-}, and S_{b1-b2+}) of subset pairs using the following equation:

$$S_{b1b2} = \frac{S_{b1+b1+} + S_{b1-b2-}}{2} - \frac{S_{b1+b1-} + S_{b1-b2+}}{2} \qquad (4)$$

The sign of Sb1b2 reflected whether the two signatures were positively or negatively correlated. The overall *P-value* (P_{b1b2}) between *b1* and *b2* was calculated using the following equation:

$$P_{b1b2} = exp\ (-|S_{b1b2}|) \qquad (5)$$

This overall *P-value* is referred to as the "overlap *P-value*" between two gene expression patterns in this paper.

REFERENCES IN SUPPLEMENTARY METHODS

1. Murano T, Hagihara H, Tajinda K, Matsumoto M, Miyakawa T. Transcriptomic immaturity inducible by neural hyperexcitation is shared by multiple neuropsychiatric disorders. Communications Biology. 2019 Jan 22;2(1):32.

2. Murano T, Koshimizu H, Hagihara H, Miyakawa T. Transcriptomic immaturity of the hippocampus and prefrontal cortex in patients with alcoholism. Scientific Reports. 2017 Mar;7:44531.

3. Hagihara H, Ohira K, Takao K, Miyakawa T. Transcriptomic evidence for immaturity of the prefrontal cortex in patients with schizophrenia. Mol Brain. 2014 May 29;7:41.

4. Ryan SD, Dolatabadi N, Chan SF, Zhang X, Akhtar MW, Parker J, et al. Isogenic Human iPSC Parkinson's Model Shows Nitrosative Stress-Induced Dysfunction in MEF2-PGC1α Transcription. Cell. 2013 Dec;155(6):1351–64.

5. Takao K, Miyakawa T. Genomic responses in mouse models greatly mimic human inflammatory diseases. PNAS. 2015 Jan 27;112(4):1167–72.

6. Kupershmidt I, Su QJ, Grewal A, Sundaresh S, Halperin I, Flynn J, et al. Ontology-based meta-analysis of global collections of high-throughput public data. PLoS ONE. 2010 Sep;5(9):e13066.
